# Large Numbers for Small Children—Up to What Age Do Infants Benefit from a Longer Echo Time in Cerebral T2 MRI Sequences?

**DOI:** 10.3390/children11050511

**Published:** 2024-04-24

**Authors:** Anne Bettina Beeskow, Franz Wolfgang Hirsch, Timm Denecke, Ina Sorge, Daniel Gräfe

**Affiliations:** 1 Department for Diagnostic and Interventional Radiology, University Hospital Leipzig, Liebigstrasse 20a, 04103 Leipzig, Germany; timm.denecke@medizin.uni.leipzig.de; 2 Department for Pediatric Radiology, University Hospital Leipzig, Liebigstrasse 20a, 04103 Leipzig, Germany; franzwolfgang.hirsch@medizin.uni-leipzig.de (F.W.H.); ina.sorge@medizin.uni-leipzig.de (I.S.); daniel.graefe@medizin.uni-leipzig.de (D.G.)

**Keywords:** MRI, brain/brain stem, infant, newborn, echo time, Michelson contrast

## Abstract

In newborns, white matter shows a high T2-weighted (T2w) signal in MRI with poor grey–white matter contrast. To increase this contrast, an extremely long echo time (TE) is used in the examination of children. It is not known up to what age this long TE should be used. The purpose of this study was to find up to what age a long TE should be used in infants. In the prospective study, 101 infants (0–18 months) underwent cranial MRI at 3 Tesla. T2-weighted Fast Spin Echo sequences with long TE (200 ms) and medium TE (100 ms) were used. The signal intensities of the cortex and white matter were measured and the grey–white matter contrast (MC) was calculated. A cut-off age was determined. The T2w sequences with long TE had a statistically significantly higher MC until the age of six months (medium TE: 0.1 ± 0.05, Long TE: 0.19 ± 0.07; *p* < 0.001). After the tenth month, the T2w sequence with medium TE provided significantly better MC (Medium TE: 0.1 ± 0.05; long TE: 0.05 ± 0.4; *p* < 0.001). The use of a long TE is only helpful in the first six months of life. After the tenth month of life, a medium TE should be favored as is used in adult brain MRI.

## 1. Introduction

Neonates and infants represent a special group in regards to the grey-white matter differentiation in cerebral imaging. Compared to adults, newborns have a higher water content and lower lipid content in their white matter due to incomplete myelination [1,2]. The axon myelination in the white matter begins before birth [2] and increases rapidly during the first two years of life and continues even up to the third decade of life [3].

The different percentage composition of white matter in newborns results in distinct features in MRI when compared to those of adults. The modified T2w signal originates from the hydrogen protons situated amidst the lipid layers of myelin. As myelination is still in its early stages, meaning that the lipid content is low and the water content is high, this leads to an increased T2-weighted signal. 

**Figure 1:**
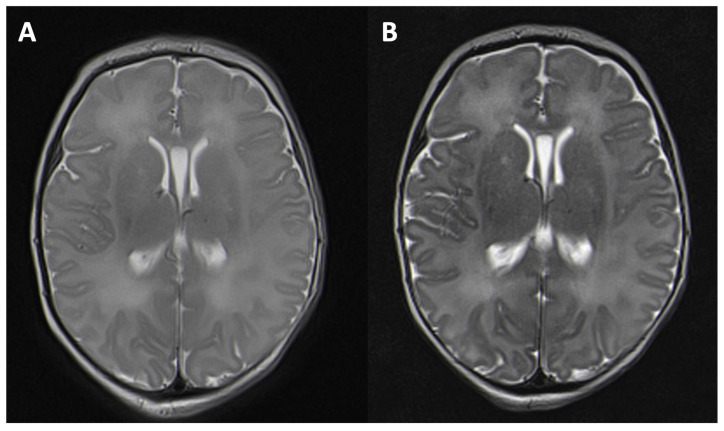
T2-weighted image in an axial orientation of a six-day-old girl after asphyxia. A higher grey–white matter contrast can be seen with a longer echo time of 203 ms, repetition time of 5500 (**B**) than with a medium echo time of 113 ms, repetition time of 6950 (**A**).

**Figure 2:**
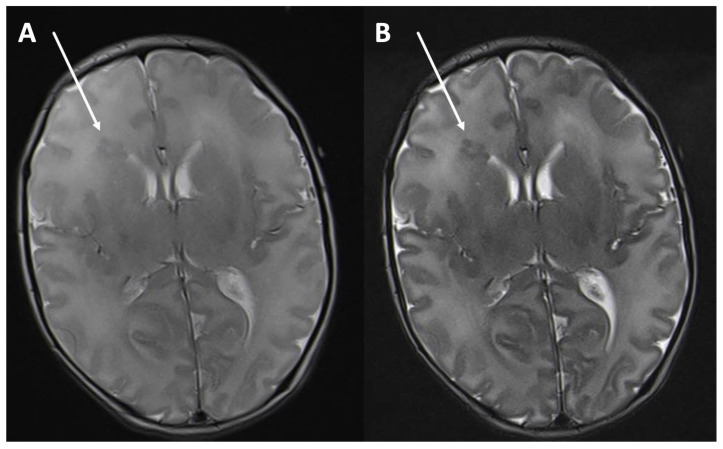
T2-weighted image in an axial orientation of a two-week-old male with seizures. The periventricular right frontal heterotopia (arrow) can be seen more clearly with a longer echo time of 203 ms, TR 5500 (**B**) than with a medium echo time of 113 ms, TR 5500 (**A**).

In detail, the posterior limb of the internal capsule is the first to show a similar T2w signal to that in adults (birth–2nd month of life) [1,2]. This is followed by the myelination of the cerebellum and the corpus callosum. Starting from the 9th month of life, the T2w signal of the white matter begins to resemble that of adults [2,3]. Myelination progresses from the occipital to the frontal areas. After 18 months of life, the white matter demonstrates almost the same T2w signal as that in adults [2,3,4]. In MRI, the myelinization of the last medullary structures (perifocal to the anterior frontal lobes) can be observed by the age of two or three years [2,3]. Due to the low content of myelin and the high content of water, T2w imaging is the most useful way of depicting myelination in infants [5]. Contrary to that of adults in a T2w sequence, the cortex of newborns is more hypointense than their white matter. During the first year of life, an inversion of contrast can be observed. As this process runs through a period of grey-white-matter isointensity, MRI diagnosis is hindered during this stage. Overall, in neonates, the cortex and white matter have a high T2w signal, which provides a low contrast between the grey and the white matter. This is of particular importance in neonates after asphyxia and in questions of medullary oedema as well as in identifying malformations in cerebral cortical development, which can be difficult to assess. In infants, this problem also occurs in the context of delayed myelinization and suspected metabolic disorders. Several technical parameters have an impact on the grey–white matter signal in T2w MRI: the repetition time, echo spacing, receiver bandwidth, flip angle, and echo train length. However, the most relevant parameter of increasing grey–white matter contrast is the TE [6,7] due to the high water content of white matter. With a long TE, a more T2w image can achieve a higher grey–white matter contrast (Figure 1A,B). In addition, the pathologies of the grey–white matter can be displayed more sharply (Figure 2A,B). As myelination progresses in the first year of life, an inversion in the grey–white matter contrast can be observed, which, in turn, leads to a bright cortex and dark white matter, as can also be seen in adults. TE in standard T2w sequences varies between manufacturers and field strength. In adults, a TE of around 101 ms at 3T is recommended [8], whereas in infants, a longer TE of about 200 ms has been proposed [9,10]. However, the literature does not specify up to which month of life a longer TE is beneficial. The aim of this study is to determine a threshold for the use of a longer TE in infants. 

## 2. Materials and Methods

### 2.1. Patients

**Figure 3:**
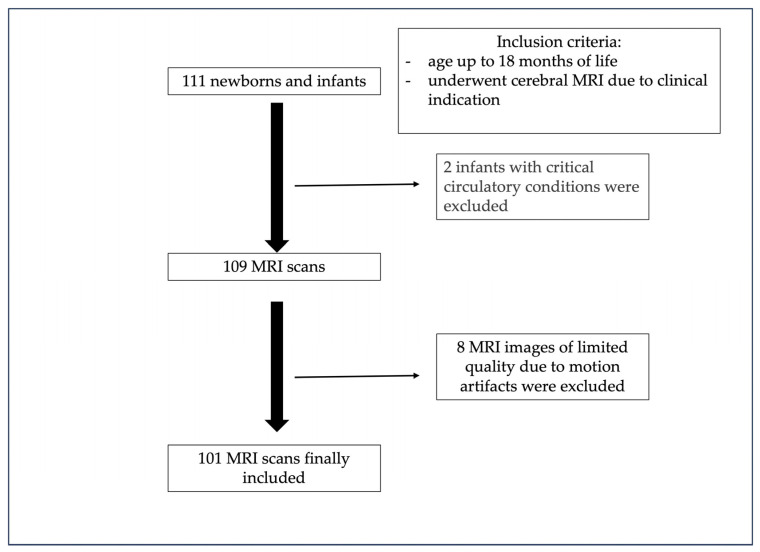
Patient inclusion flowchart.

Between June 2021 and January 2023, all newborns and infants between six days and 18 months of age (n = 111) who underwent cerebral MRI for clinical indication were prospectively enrolled in our study (Figure 3). The approval of the ethical committee and written consent from the parents or representatives was obtained for this study. In the children older than one month, MRI was performed under sedation. Newborns below four weeks of age were examined without sedation after feeding, using a vacuum mattress for immobilization. All newborns and infants who received an MRI for a clinical indication were included. Each MRI examination was planned and performed with at least a T2 spin echo sequence with a medium and a long TE. The other sequences differed depending on the clinical symptoms and were not evaluated in our study.

Patient characteristics are shown in Table 1. The examinations were carried out for various indications such as the question of intracranial abnormalities in seizures (n = 38), the suspicion of syndromic disease (n = 19), developmental delays (n = 19), after asphyxia (n = 17), after intracranial bleeding (traumatic/peri/neonatal; n = 15), macrocephalus (n = 14), suspected congenital malformations (n = 12), suspected Horner’s syndrome (n = 2), suspected shaken baby syndrome (n = 4) and suspected meningitis (n = 4). Two infants with critical circulatory conditions were excluded so as not to prolong the planning and examination time.

After the examination, the images with limited quality due to motion artifacts in one of the sequences were excluded.

### 2.2. MRI Protocol

All studies were performed on a 3T MRI scanner (TrioTim or Prisma fit, Siemens, Erlangen, Germany) using a 32- or 63-channel head. The MRI protocol included at least one T2w spin echo sequence, one T1 3D isovoxel dataset and one diffusion-weighted sequence for all examinations. In all children examined with the TrioTim MRI (see Table 1 for scanning parameters), a T2w fast spin echo sequence with a medium-length TE of 113 ms and a second T2w spin echo sequence with an echo time of 203 ms were acquired. All other scan parameters remained unchanged. The TE of 203 ms represented the longest TE available on this device. In all children examined with the Prisma fit scanner, the acquisition was performed as a double echo readout, T2w, fast spin echo sequence (scanning parameters see Table 2). 

### 2.3. Quantitative MRI Analysis

The sequences were reviewed by three readers with 5 (ABB), 15 (DG) and 24 (IS) years of experience in pediatric cerebral MRI. They assessed whether there were unremarkable findings or pathologies of the grey or white matter. Signal intensities (SIs) were measured in an axial orientation supratentorial at the basal ganglia level and infratentorial at the cerebellar nuclei level. A region of interest of approximately 3 mm^2^ was manually drawn in the following localizations: the right and left frontal cortex and the adjoining white matter, the right and left occipital cortex and the adjoining white matter. Care was taken to ensure that only grey matter and no cerebrospinal fluid was present in the cortex ROI. This was done for both the T2w sequences with long and with intermediate TE with the selection of the equivalent areas in each patient with a standard DICOM Viewer (Intellispace Portal v12, Philips, Best, The Netherlands). The resulting SIs were used to determine the Michelson contrast (MC) as a suitable tool to express differences in intensity between bright and dark structures (8,9). The equation for MC was MC = ((*S*I*max* − *S*I*min*))/((*S*I*max* + *S*I*min*)), with MC ≤ 1. Interobserver and intra-observer variability was evaluated by means of the intraclass correlation coefficient (ICC). 

For a statistical comparison between standard TE and long TE dependent on age, small groups were first formed at two-month intervals. Subsequently, groups with significant differences were summarized into a total of three groups (0–6 months, 6–10 months, 10–18 months) and a statistical test with a subsequent Bonferroni correction for multiple comparisons was carried out in these three groups. The normal distribution of the data was evaluated using the Shapiro–Wilk test. The Wilcoxon signed-rank test was employed to compare the central tendencies of the groups.

The statistical evaluation was carried out using IBM SPSS Statistics 27 (IBM, New York, NY, USA). A 95% confidence interval with a significance level of *p* < 0.05 was chosen. 

## 3. Results

### 3.1. Cohort

**Figure 4:**
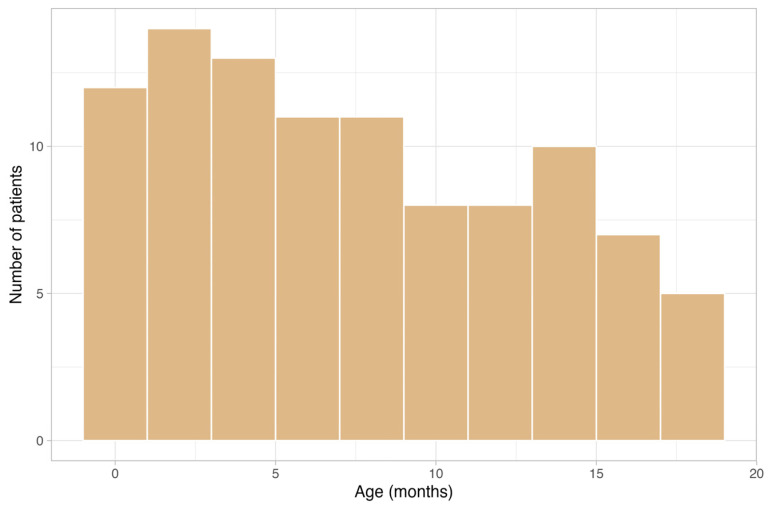
The age distribution of the patients included.

In total, 111 infants were examined using cerebral MRI. Two infants were excluded due to critical cardiocirculatory conditions and eight children were excluded due to motion artifacts. Therefore, 101 children aged between six days and 18 months were included (median age = 6.4 months, interquartile range 2.9–11.8, 54 males) (Figure 4). Among the children, six were preterm babies (11%). One baby was examined six days after birth at a corrected gestational age of 34 + 4 weeks, and the other preterm babies were scanned at a corrected age of two (n = 3), five and eleven months. 

### 3.2. Michelson Contrast

**Figure 5:**
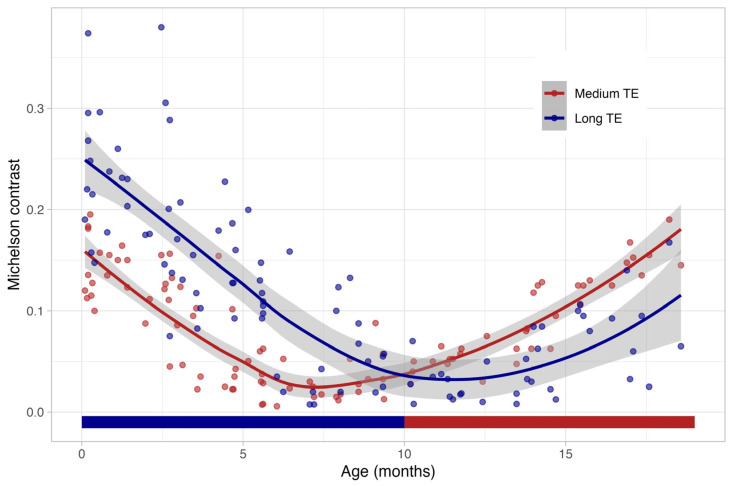
The course of the cerebral Michelson contrast (shown as a magnitude) between birth and 20 months in T2 fast spin echo sequences with a long (blue) and a medium (red) echo time (local polynomial regression fitting with the 95% confidence interval as the shaded area). Until about six months, the long TE provides a better contrast between the grey and the white matter. After the tenth month, in adolescence and in adulthood, the shorter echo time is superior.

In Figure 5, the course of the cerebral Michelson contrast is shown between birth and 18 months in T2 FSE sequences with long (blue) and medium (red) echo times. Until about six months, the long TE provides a better contrast between the grey and the white matter. After the tenth month, as well as in adolescence and adulthood, the shorter echo time is superior.

The mean MC of the standard TE was 0.10 (±0.05), while the mean MC of the long TE was 0.19 (±0.07) (Table 3). From seven to nine months of age, no significant difference in MC was observed for both TEs. During this period, an inversion of the contrast from the dark cortex and the bright white matter to the bright cortex and the dark white matter also took place with a generally low MC: the mean MC of the medium TE was 0.01 (±0.03) and the mean MC for the long TE was 0.01 (±0.08). After the tenth month of life, a long TE yielded a higher MC compared to the standard medium-long TE: the mean MC for the medium TE was 0.10 (±0.05), whereas the mean MC for the long TE was 0.05 (±0.04). The mean ICC was moderate (0.51 for interobserver variability, and 0.67 for intra-observer variability).

## 4. Discussion

The higher contrast between grey and white matter in the T2w sequences with a long TE in this age group is mainly caused by the low myelination in newborns. It is known that this higher content of fluid can be better displayed with a stronger T2-weighted image. Myelination, synaptogenesis and synaptic pruning progress significantly during the first year of life and result in an increased concentration of macromolecules [11,12]. Consequently, a generalized reduction in free water content [4,13] takes place and results in a decreased intensity in the T2w sequence. 

This study aimed to find up to what age a long TE should be used in neonates and infants. Our data showed that a long TE is only beneficial until the age of six months. Since grey–white matter contrast in T2w sequences with long and medium TEs converge after six months of life (Figure 4), there is no definite advantage of a long TE (203 ms) over a medium TE (113 ms). During the period from the 7th to the 9th month of life, there was no difference in the grey and white matter contrasts between the long and medium-long TE periods. However, the T2w contrast in this age group presents a diagnostic challenge due to a blind window for TE. After the 10th month of life, the medium-long TE (113 ms), which is used for adults, showed a higher grey and white matter contrast. These findings align with the understanding of progressive white matter myelination. Like Barkovich et al. [3] demonstrated, between 9 and 11 months of age, the T2-weighted signal becomes similar to that of adults due to the progressive myelination from the occipital to the frontal region.

The visualization of progressive and different myelination is the subject of several recent publications. This shows how important this topic is in the cerebral imaging of children. There are attempts to optimize the visualization of progressive myelination and brain areas of different myelination [10,14,15], for instance with a T1w/T2w ratio, spectroscopy [16] or fMRI [1,12,17]. These studies also attempt to improve visualization by changing the TE. In this context, extended TEs in the T2* sequence are also favored [1,4]. Using a long TE in young children is a well-known piece of advice already being implemented in many hospitals [11,13,16]. However, there is still disagreement as to when a long TE should be used. Several studies indicate that optimal MRI parameters (the TE, TR and T1/T2 ratio) are age-dependent [14,18,19]. Jones et al. determined the optimal TR in 10 newborns to be 1712 +/− 235 ms, compared to more than 3000 ms in adults [14]. Concordantly, Soun et al. showed that the T1/T2 ratios of 10 newborns differ from those of adults due to their different myelination [12]. Welker et al. [18] recommended an evaluation of the MRI images of children always with regard to the landmarks of progressive myelination. A recommendation for the exact examination parameters for children between 1 month and 2 years of age has not yet been made. Our study found a significantly higher MC in a long TE when compared to a medium TE in the age group below six months. 

A long TE is already used in many centers, but there is a lack of data on the age up to which a long TE should be used. An increased contrast of white and grey matter is beneficial in everyday clinical practice to detect pathologies more reliably. In particular, white matter edemas can otherwise be quite challenging to detect, especially white matter edemas in brains with low myelination. Another pathology, obviously more readily depicted with an improved gray-matter-to-white-matter contrast, is that of heterotopias. 

Similarly, variations in the optimal TEs across different brain regions have also been reported [20] with longer values in deeper brain regions compared to those of the more superficial gyrus. Basal ganglia TEs were not measured in our study but could be the subject of further investigation.

A limitation of this study was the moderate sample size. In addition, two different MRI scanners with slightly different TEs were used. The ICC for the MC was only moderate, indicating that the MC also varies between neighboring regions. Besides TE, there are several MRI parameters [6] that influence the grey–white matter contrast in T2w sequences, such as the repetition time, echo spacing, receiver bandwidth, flip angle, echo train length and voxel size. In order to be able to investigate the influence of the TE, all other scan parameters were left constant. 

In conclusion, at up to six months of age, a long TE is preferable. From the tenth month of age, a medium TE should be used as it is in adulthood. 

## Figures and Tables

**Table 1:** Patient characteristics.

Patient Characteristics	
Mean Age (months) Minimum Maximum	7.8 6 days 18 months
Sex Male Female	n = 54 n = 46
Birth on schedule Preterm	n = 94 n = 7
Reason of scanning - Seizures - Suspicion of syndromic disease - Developmental delay - Status post asphyxia - Status post intracranial bleeding - Macrocephalus - Suspected congenital malformation - Suspected meningitis - Suspected shaken baby syndrome - Suspected Horner’s syndrome	n = 38 n = 19 n = 19 n = 17 n = 15 n = 14 n = 12 n = 4 n = 4 n = 2
Normal findings in MRI	n = 51

**Table 2:** The technical parameters of the T2-weighted sequences employed. Depending on the 3T scanner, single readout or double readout approaches were used.

Scanning Parameters	Dual TE (n = 71)	Single TE (n = 30)
TE—Echo time (ms)	100/200	113/203
TR—Repetition time (ms)	6950	5500
Echo-train length	7	12
Acquisition matrix	384 × 237	320 × 320
Number of excitations	2	1
Bandwidth (Hz/px)	221	363
Field of view	180 × 146	180 × 180
Slice thickness (mm)	3	3
Number of slices	40	40
Flip angle (°)	150	90
Scanning time (m:ss)	3:30	2:17/2:25

**Table 3:** The Michelson contrasts of medium TE and long TE in different age groups (* = statistically significant). TE: Echo time; CI: Confidence interval; MC: Michelson contrast.

Age (Months)	Sample Size	Medium TE (MC)	Long TE (MC)	95% CI		*p*-Value
				Lower	Upper	
0–6	47	0.11	0.18	−0.10	−0.07	<0.001 (*)
6–10	18	−0.02	−0.01	−0.05	0.00	0.24
10–18	34	−0.10	−0.04	0.03	0.05	<0.001 (*)

## Data Availability

The data presented in this study are available on request from the corresponding author. The data are not publicly available due to ethical restriction.
